# Short- and long-term outcome of patients with spontaneous echo contrast or thrombus in the left atrial appendage in the era of the direct acting anticoagulants

**DOI:** 10.1007/s00392-021-01926-8

**Published:** 2021-08-26

**Authors:** Julian Felix Backhaus, Andreas Pflaumbaum, Christos Krogias, Fabienne Kreimer, Andreas Mügge, Ralf Gold, Michael Gotzmann

**Affiliations:** 1 Cardiology and Rhythmology, University Hospital St. Josef Hospital, Ruhr-University Bochum, Gudrunstraße 56, 44791 Bochum, Germany; 2 Neurology, St. Josef-Hospital, Ruhr-University Bochum, Gudrunstraße 56, 44791 Bochum, Germany

**Keywords:** Thrombi and spontaneous echo contrast, Outcome, Direct acting anticoagulants

## Abstract

**Background:**

Thrombi and spontaneous echo contrast (SEC) in the left atrial appendage (LAA) are associated with thromboembolic events and poor prognosis. There are very few data on long-term outcome, especially with the use of direct acting anticoagulants (DOAC).

**Methods:**

In this retrospective study, all transoesophageal echocardiographies performed at a tertiary care university hospital from 2015 to 2020 were analyzed. All patients with thrombus or SEC in the LAA were included. Medical history, laboratory, echocardiographic parameters and medication at discharge were documented. The primary endpoint of the study was a composite endpoint (all-cause mortality, non-fatal stroke or transient ischaemic attack [TIA], non-fatal systemic embolization, non-fatal major bleeding and non-fatal myocardial infarction).

**Results:**

Of a total of 4062 transoesophageal echocardiographies, thrombi were detected in 51 patients (1.2%) and SEC in 251 patients (6.2%). These patients formed the final study cohort (*n* = 302). During a mean follow-up period of 956 ± 663 days, 87 patients (29%) suffered the primary point. The following baseline characteristics predicted the primary endpoint: age, haemoglobin, a previous coronary artery bypass grafting, dialysis and choice of anticoagulation. Prescription of apixaban at discharge was associated with lower rate of adverse events (hazard ratio 0.564, confidence interval 0.331–0.960; *p* = 0.035) while prescription of dabigatran was associated with higher rate of adverse events (hazard ratio 3.091, confidence interval 1.506–6.347; *p* = 0.002).

**Conclusion:**

Even in the DOAC era, the occurrence of thrombus or SEC in the LAA is associated with a high rate of MACCE. Our study suggests that the choice of DOAC therapy may have an impact on long-term survival.

**Graphic abstract:**

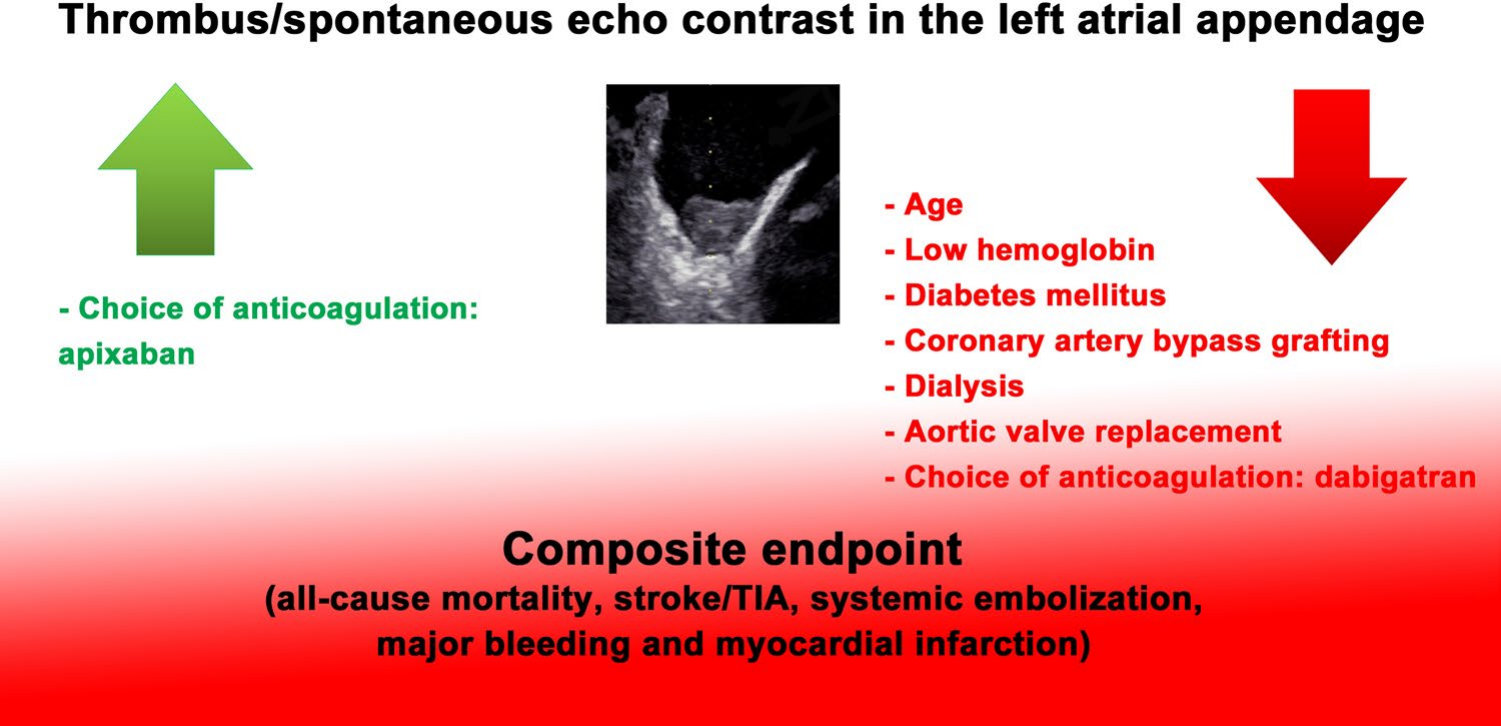

**Supplementary Information:**

The online version contains supplementary material available at 10.1007/s00392-021-01926-8.

## Introduction

Thrombi and spontaneous echo contrast (SEC) in the left atrial appendage (LAA) are relatively common findings on transesophageal echocardiography [1]. Both abnormalities are associated with thromboembolism and poor prognosis [2–7]. Thrombi and SEC in the LAA occur predominantly in atrial fibrillation (AF) but have also been described in patients without AF [8].

The detection of a thrombus in the LAA is usually an indication for therapeutic anticoagulation [9]. In contrast, in the case of an SEC, the recommendation for anticoagulants is only given in the case of corresponding pre-existing conditions (predominantly atrial fibrillation with increased thromboembolic risk) [10]. About 10 years ago, the direct acting anticoagulants (DOACs) were introduced for the prophylaxis of thromboembolic events in patients with AF [11–14]. Despite the partial lack of data from prospective randomised controlled trials [15], DOACs are used in many centers for the treatment of thrombi in the LAA [16].

However, there are few data on the long-term outcome of patients with thrombus or SEC in the LAA on anticoagulation. In particular, there are very scarce studies on anticoagulation with DOAC in this group of patients [17–19].

Therefore, the aims of this study were (1) to investigate the prevalence of thrombus and SEC in the LAA in the DOAC era, (2) to analyze the long-term outcome in terms of death, stroke, major bleeding, systemic embolization, and myocardial infarction of patients with thrombus and SEC, and (3) to investigate the impact of anticoagulation on event-free long-term survival.

## Methods

This study examined all patients in the years 2015 to 2020 in whom a thrombus or SEC was detected in LAA on transoesophageal echocardiography. All examinations were performed at the St. Josef Hospital—Hospital of the Ruhr University Bochum. The indications for transoesophageal echocardiography were as follows: planned electrical or pharmacological cardioversion, prior to pulmonary vein isolation or ventricular tachycardia ablation, prior to left atrial appendage occlusion, in cases of cryptogenic stroke, TIA or other thromboembolic events, for evaluation of heart valves, unclear infection or suspected aortic dissection. Patients gave informed consent.

Medical history, laboratory examinations (creatinine and hemoglobin), echocardiography results, medication before and after transoesophageal echocardiography were recorded for all patients. This study is a retrospective analysis. The study was approved by the local ethics committee of the Ruhr University Bochum (Number 21-7234-BR).

### Inclusion and exclusion criteria and endpoint

For this study, we examined retrospectively all transoesophageal echocardiographies performed between 2015 and 2020. All patients in whom a thrombus or a SEC in the LAA was detected were included in this analysis. Patients with thrombi in other cardiac cavities, cardiac tumours and previous interventional or surgical occlusion of the LAA were excluded.

The primary endpoint of the study was a composite endpoint (all-cause mortality, non-fatal stroke or transient ischaemic attack [TIA], non-fatal systemic embolization, non-fatal major bleeding and non-fatal myocardial infarction). Major bleeding was defined according to the Bleeding Academic Research Consortium criteria as BARC ≥ 3 [20]. For the analyses of the combined primary endpoint, the time to the occurrence of the first adverse event was considered. Secondary study endpoint was all-cause mortality. We obtained the follow-up information based on outpatient and inpatient admissions. In addition, a follow-up was performed in 2021, either in personal or telephone contact or with the deceased patients' general practitioners.

### Echocardiography

Transthoracic echocardiography was performed within 7 days before transoesophageal echocardiography. Both examinations were conducted with a digital ultrasound scanner (Vivid E9; General Electric, Horton, Norway). Transoesophageal echocardiography was performed by experienced examiners according to current recommendations [21].

A thrombus in the LAA was defined as a solid echo-dense structure detectable in multiple imaging planes in the LAA [22]. If a thrombus was detected, transoesophageal echocardiography was repeated at least 3 weeks after the initial examination [9].

SEC in the LAA was diagnosed with evidence of swirling dynamic echoes without evidence of thrombus. In accordance with the EACVI/EHRA recommendation, SEC was classified as mild to moderate SEC (swirling pattern in the LAA), severe SEC (intense echo-density and very slow swirling patterns in the LAA) and sludge (dense smoke, viscid echo-density, not solid) [1]. These patients underwent no routine repeated transoesophageal echocardiography. In the case of simultaneous occurrence of thrombus and SEC in the LAA, only the thrombus was assessed for the calculations.

### Anticoagulation

Anticoagulation was continued or initiated in all patients in whom a thrombus was detected. Patients who had not previously received anticoagulation were given intravenous heparin immediately. In patients on vitamin K antagonist (VKA) therapy with effective INR values or on DOAC therapy, this therapy was continued or changed according to the clinical decision of the treating physician.

In patients with SEC, anticoagulation was prescribed if the patient had AF with a CHADS-VASc score of ≥ 1 or if there were other reasons for anticoagulation (e.g. mechanical valve replacement, pulmonary embolism or deep vein thrombosis). Acetylsalicylic acid and P2Y12 inhibitors and combinations were used due to patient comorbidities and not directly for thrombus or SEC therapy. The documented anticoagulation medication reflects the medication at the time of discharge after initial transoesophageal echocardiography.

### Statistics

Numerical values are expressed as mean ± standard deviation. Continuous variables were compared between groups using an unpaired *t* test (for normally distributed variables) or Mann–Whitney U test (for non‐normally distributed variables). *χ*^2^ analysis was used to compare categoric variables. All variables in Tables 1, 2 and 3 were evaluated for the primary end point and all variables in Tables supplement 1, 2 and 3 were evaluated for the secondary endpoint in a univariate and multivariate Cox proportional hazard model to identify independent predictors of outcome. Receiver operating characteristic curves were generated to define cutoff values for independent predictors’ age and hemoglobin. Results are present as hazard risk. Freedom from primary and secondary endpoints was analyzed by the Kaplan–Meier method and curves were compared by the log‐rank test. A *P* value < 0.05 was considered significant. All probability values reported are 2‐sided.

**Table 1:** Baseline Characteristics in patients with and without a primary endpoint (*n* = 302)

	Primary endpoint (*n* = 87)	No primary endpoint (*n* = 215)	*p* value
Age (years)	75.1 ± 7.6	70.7 ± 10.2	< 0.001
Women (♀), *n* (%)	38 (44)	95 (44)	0.936
Medical history			
Hypertension, *n* (%)	81 (93)	195 (91)	0.500
Diabetes mellitus, *n* (%)	39 (45)	55 (26)	0.001
Atrial fibrillation, *n* (%)	82 (94)	209 (97)	0.214
Coronary artery disease, *n* (%)	37 (43)	61 (28)	0.017
Previous myocardial infarction, *n* (%)	14 (16)	26 (12)	0.353
Coronary artery bypass grafting, *n* (%)	13 (15)	5 (2)	< 0.001
Previous stroke/TIA, *n* (%)	24 (28)	34 (16)	0.020
Peripheral artery disease, *n* (%)	17 (20)	24 (11)	0.054
Chronic obstructive lung disease, *n* (%)	12 (14)	21 (10)	0.310
Heart failure, *n* (%)	40 (46)	61 (28)	0.003
ICD/CRT, *n* (%)	12 (14)	8 (4)	0.001
Dialysis, *n* (%)	6 (7)	0	< 0.001
CHA2DS2-VASc Score (pts)	4.75 ± 1.67	3.69 ± 1.71	< 0.001
Labor			
Hemoglobin (g/dL)	12.4 ± 2.3	13.8 ± 1.8	< 0.001
Creatinine (mg/dL)	1.33 ± 1.2	1.05 ± 0.35	0.002

*TIA* transient ischaemic attack, *ICD/CRT* implantable cardioverter defibrillator/cardiac re-synchronisation therapy

**Table 2:** Transthoracic and transoesophageal echocardiographic parameters of study patients (*n* = 302)

	Primary endpoint (*n* = 87)	No primary endpoint (*n* = 215)	*p* value
Left ventricular ejection fraction (%)	47.7 ± 12.1	48.5 ± 11.6	0.579
Left atrial diameter (mm)	45.3 ± 6	43.9 ± 5.4	0.064
Ventricular septum thickness (mm)	12.6 ± 2.1	12.2 ± 2	0.155
Left atrial appendage thrombus, *n* (%)	20 (23)	31 (14)	0.072
Spontaneous echo contrast (mild to moderate, severe, sludge)	47/17/3	147/35/2	0.111
Aortic stenosis (none/ mild/ moderate/ severe), *n*	81/2/2/2	204/3/3/5	0.808
Aortic regurgitation (none/ mild/ moderate/ severe), *n*	42/42/1/2	126/82/2/0	0.063
Mitral stenosis (none/ mild/ moderate/ severe), *n*	85/1/1/0	208/4/1/2	0.696
Mitral regurgitation (none/ mild/ moderate/ severe), *n*	6/71/8/2	23/155/36/1	0.216
Tricuspid regurgitation (none/ mild/ moderate/ severe), *n*	22/48/14/3	75/120/17/3	0.068
Aortic valve replacement/TAVI, *n* (%)	7 (8)	4 (2)	0.009
Mitral valve repair/ replacement *n* (%)	3 (3)	7 (3)	0.933

*TAVI* trans-aortic valve implantation

**Table 3:** Medication at discharge after initial transoesophageal echocardiography of study patients (*n* = 302)

	Primary endpoint (*n* = 87)	No primary endpoint (*n* = 215)	*p* value
Acetylsalicylic acid, *n* (%)	20 (23)	37 (17)	0.245
P2Y12-Inhibitor, *n* (%)	2 (2)	14 (7)	0.139
Dual therapy*, *n* (%)	14 (16)	25 (12)	0.295
Triple therapy*, *n* (%)	0	10 (5)	0.041
Vitamin K antagonist, *n* (%)	34 (39)	63 (29)	0.099
Apixaban, *n* (%)	21 (24)	110 (51)	< 0.001
Dabigatran, *n* (%)	10 (11)	9 (4)	0.018
Edoxaban, *n* (%)	6 (7)	13 (6)	0.783
Rivaroxaban, n (%)	2 (2)	12 (6)	0.219
Heparine, *n* (%)	8 (9)	4 (2)	0.003
No anticoagulation, *n* (%)	6 (7)	4 (2)	0.027

*Dual therapy, combination of Acetylsalicylic acid/P2Y12 and DOAC/ Vitamin K antagonist, *Triple therapy, combination of Acetylsalicylic acid, P2Y12 and DOAC/ Vitamin K antagonist. DOAC and a Vitamin K antagonist are not used concomitantly

## Results

Between 2015 and 2020, 4062 transoesophageal echocardiographies were performed at St-Josef-Hospital Bochum. In 51 patients (1.2%) thrombi in the LAA was found and SEC in the LAA in 251 patients (6.2%). SEC was mild to moderate in 194 patients, severe in 52 patients and sludge was present in 5 patients. Of the 51 patients with thrombus, a total of 22 patients also had some degree of SEC. These patient subgroups did not differ in baseline characteristics or outcome. The patients with thrombus or SEC in the LAA formed the final study (*n* = 302) cohort. The mean age of the patients (44% women, 56% men) was 72 ± 9.7 years (minimum 40 years, maximum 91 years). The mean left ventricular ejection fraction was 48.3 ± 11.8% and the CHA2DS2-VASc score was 4 ± 1.8 points.

The indications for transoesophageal echocardiography in the study cohort were planned electrical (*n* = 210) or pharmacological (*n* = 2) cardioversion, prior to pulmonary vein isolation or ventricular tachycardia ablation (*n* = 27), prior to interventional left atrial appendage occlusion (n = 3), in cases of cryptogenic stroke, TIA or other thromboembolic events (*n* = 26), for evaluation of heart valves (*n* = 9), unclear infection (*n* = 18) and other indications (*n* = 7).

Patients with thrombus had a significantly lower left ventricular ejection fraction than patients with SEC (45 ± 12.4 vs. 49 ± 11.5, *p* = 0.032). Otherwise, there were no significant differences between the two subgroups of patients—in particular, there was no difference in left atrial diameter or CHA2DS2-VASc score.

Of the study patients, 291 patients (96%) had known AF or AF were diagnosed during hospitalisation (paroxysmal [*n* = 29], persistent [241], permanent [*n* = 21]). The remaining 11 patients (4%) had sinus rhythm and AF was neither known nor diagnosed. Of these, 3 patients presented with a thrombus and 8 patients with SEC.

### Treatment of patients with thrombus

Patients with thrombus detection were taking the following anticoagulation prior to transoesophageal echocardiography: no anticoagulation (*n* = 27), apixaban (*n* = 12), VKA (phenprocoumon) (*n* = 8), rivaroxaban (n = 3), heparin (*n* = 1). In some cases, medication was changed after transoesophageal echocardiography. All patients with detected thrombus received anticoagulation—either VKA, DOAC or heparin in therapeutic dosage. Most patients with thrombus received at discharge VKA therapy with a target INR of 2–3 (*n* = 25). Other medication for anticoagulation were: Apixaban (*n* = 16), Dabigatran (*n* = 6), Rivaroxaban (*n* = 1) and Heparin (*n* = 3). Of the 51 patients with thrombus in the LAA, 40 underwent repeated transoesophageal echocardiography in our hospital. In these examinations, a persistent thrombus was found in 10 patients, while the thrombus had vanished in the remaining 30 patients in the control. Of the remaining 11 patients without repeat transoesophageal echocardiography, 3 died within the first 30 days and in 8 patients, no control examination was performed.

### Treatment of patients with SEC

Patients with SEC received the following anticoagulation medication before transoesophageal echocardiography: no anticoagulation (*n* = 97), apixaban (*n* = 60), VKA (*n* = 51), rivaroxaban (*n* = 12), dabigatran (*n* = 6), edoxaban (*n* = 12) and heparin (*n* = 13). Detection of SEC usually did not lead to a change in anticoagulation. However, other conditions—particularly the initial diagnosis of AF—led to anticoagulant prescriptions. At discharge, patients with SEC were receiving the following anticoagulants: apixaban (*n* = 115), VKA (*n* = 72), rivaroxaban (*n* = 13), dabigatran (*n* = 13), edoxaban (*n* = 19), heparin (*n* = 9) and no anticoagulation (*n* = 10).

### Reduced dose of DOAC

At the time of discharge, the dose of DOAC was chosen according to the current recommendation. The reduced dose was prescribed for relevant comorbidities [23]. Of the 302 study patients, a total of 131 patients received apixaban (reduced dose: 2 × 2.5 mg, n = 18), 19 patients received dabigatran (reduced dose: 2 × 110 mg, *n* = 8), 19 patients received edoxaban (reduced dose: 1 × 30 mg, *n* = 7), and 14 patients received rivaroxaban (reduced dose: 1 × 15 mg, *n* = 0).

### Patients receiving no anticoagulation or heparin

Of the 302 study patients, a total of 10 patients (3%) with SEC did not receive anticoagulation. Three patients underwent implantation of an interventional LAA occluder and initiation of dual antiplatelet therapy. In another 3 patients with mild to moderate SEC, sinus rhythm was present without evidence of AF. Individual factors were present in the remaining patients: gastrointestinal varices, tumor disease, severe wound healing disorder and refusal of anticoagulation therapy.

In total, 12 patients (4%) received therapy only with heparin. Of these, 5 patients received only a prophylactic dose and 7 patients received a therapeutic dose of low molecular weight or unfractionated heparin. The decision to provide heparin therapy was due to concomitant conditions: immediately planned surgery (*n* = 4), prolonged treatment in the intensive care unit with subsequent death (*n* = 3), recurrent bleeding (*n* = 1), active tumor disease (*n* = 1), refusal of oral anticoagulation for dementia (*n* = 1) and sinus rhythm without thrombus (*n* = 2).

### Adverse events

The following adverse events occurred in the study population during the entire observation period: (1) Fatal events: Death from any cause (*n* = 70). The causes of death were stroke (*n* = 5), myocardial infarction (*n* = 2), heart failure (*n* = 7), sudden cardiac death (*n* = 4), acute vascular occlusion (*n* = 1), tumour disease (*n* = 3), renal failure (*n* = 2), sepsis (*n* = 10) and unclear or multiple causes (*n* = 36). 2) In addition, the following non-fatal events occurred: non-fatal stroke/TIA (*n* = 16; of which ischaemic stroke, *n* = 15, intracerebral bleeding *n* = 1), non-fatal embolisation (*n* = 2), non-fatal severe bleeding *n* = 10; of which gastrointestinal bleeding, *n* = 6, intracerebral bleeding *n* = 1, other *n* = 3), non-fatal myocardial infarction (*n* = 1).

### Primary endpoint

**Fig. 1:**
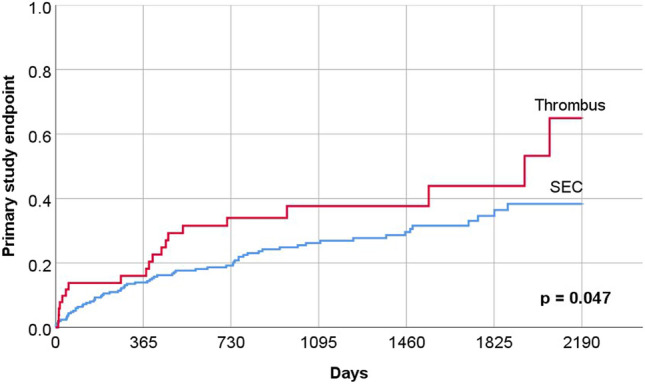
Short- and long-term outcome (primary endpoint) in study patients according to presence of thrombus or spontaneous echo contrast in left atrial appendage

During a mean follow-up period of 956 ± 663 days, 87 patients (29%) suffered the primary point (composite endpoint of all-cause mortality [*n* = 61], non-fatal stroke or TIA [*n* = 16], non-fatal systemic embolization [*n* = 1], non-fatal major bleeding [*n* = 8] and non-fatal myocardial infarction [*n* = 1]). Clinical characteristics, echocardiographic parameters, and medication at discharge are listed in Tables 1, 2 and 3. Table 4 lists all parameters that were significantly associated with the occurrence of primary endpoint in the long-term course. Notably, the occurrence of thrombus in the LAA was associated with a significantly higher rate of primary endpoint in the long-term outcome (Fig. 1).

**Table 4:** Univariate und multivariate analysis: predictors of long-term outcome

		Univariate analysis			Multivariate analysis	
	Hazard ratio	Confidence interval	*p* value	Hazard ratio	Confidence interval	*p* value
Age (years)	1.054	1.027–1.082	< 0.001	1.036	1.006–1.066	0.019
Hemoglobin (g/dL)	0.699	0.634–0.772	< 0.001	0.762	0.682–0.852	< 0.001
Creatinine (mg/dL)	1.939	1.573–2.390	< 0.001			
CHA2DS2-VASc Score (pts)	1.374	1.217–1.551	< 0.001			
Diabetes mellitus	2.092	1.367–3.200	0.001	1.628	1.012–2.618	0.044
Heart failure	1.991	1.305–3.036	0.001			
Coronary artery disease	1.607	1.050–2.459	0.029			
Coronary artery bypass grafting	4.252	2.344–7.712	< 0.001	2.861	1.488–5.504	0.002
Previous stroke/TIA	2.074	1.294–3.324	0.002			
Dialysis	19.713	8.056–48.235	< 0.001	6.446	1.775–23.409	0.005
Aortic valve replacement	3.266	1.505–7.088	0.003	2.394	1.052–5.449	0.037
ICD/CRT	3.521	1.903–6.514	< 0.001			
Heparine	6.514	3.090–13.730	< 0.001			
Apixaban	0.411	0.251–0.673	< 0.001	0.564	0.331–0.960	0.035
Dabigatran	2.624	1.353–5.088	0.004	3.091	1.506–6.347	0.002
No anticoagulation	3.900	1.689–9.009	0.001			

*TIA* transient ischaemic attack, *ICD/CRT* implantable cardioverter defibrillator/cardiac re-synchronisation therapy

Independent parameters for the occurrence of primary endpoint in the long-term course were age, haemoglobin, diabetes mellitus, a previous coronary artery bypass grafting, dialysis and use of dabigatran and apixaban. Prescription of dabigatran at discharge was associated with higher rate of primary endpoint while prescription of apixaban was associated with a lower rate of primary endpoint (Table 4).

Using receiver operating characteristic analysis, cutoff values for separating study patients were age ≥ 70 years (area under the curve [AUC] 0.614, CI 0.547–0.618, *p* = 0.001; hazard ratio 2.176, confidence interval 1.308–3.623; *p* = 0.003) and hemoglobin < 12 mg/dl (AUC 0.683, CI 0.617–0.750, *p* < 0.001; hazard ratio 3.823, confidence interval 2.464–5.933; *p* < 0.001).

**Fig. 2:**
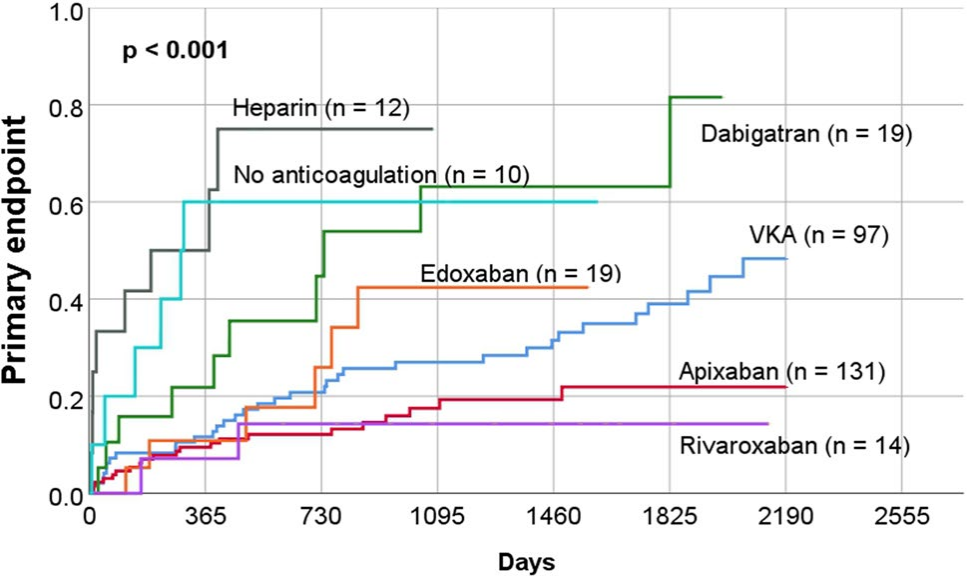
Long-term outcome (primary endpoint) in the study patients according to oral anticoagulation at the time of discharge after initial transoesophageal echocardiography

Kaplan–Meier curves were generated for primary endpoint based on the anticoagulation prescribed at the time of discharge (VKA, apixaban, dabigatran, edoxaban, rivaroxaban, heparin and no anticoagulation). This revealed a significant difference in the rates of primary endpoint between groups (*p* < 0.001) (Fig. 2).

### Secondary endpoint

**Fig. 3:**
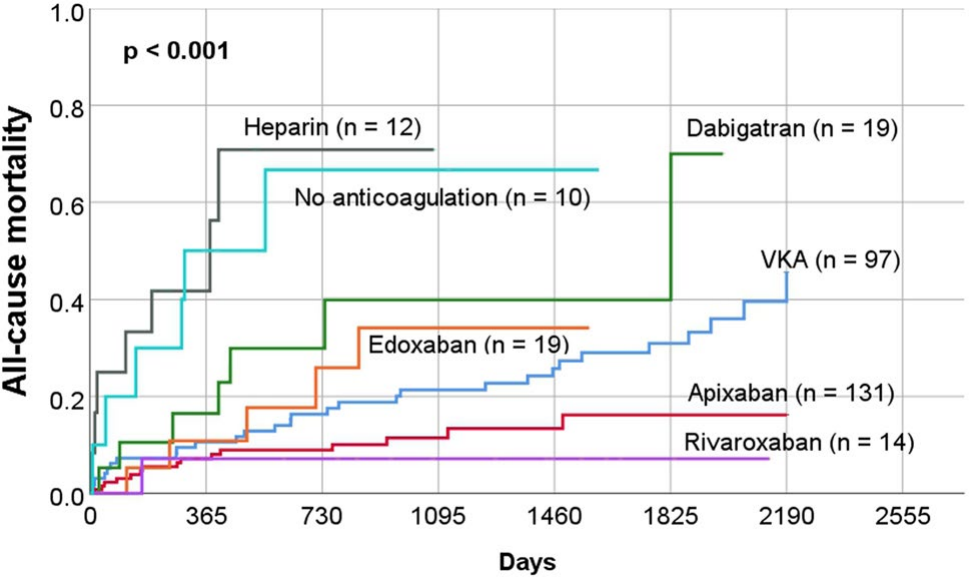
All-cause mortality (secondary endpoint) of the study patients according to oral anticoagulation at the time of discharge after initial transoesophageal echocardiography

During a mean follow-up period of 1002 ± 669 days, 70 patients (23%) suffered the secondary endpoint (all-cause mortality). Clinical characteristics, echocardiographic parameters, and medications are listed in tables (Tables Supplement 1, 2 and 3). Table Supplement 4 lists all parameters that were significantly associated with the secondary study endpoint. Multivariate analysis showed that the parameters age, haemoglobin, creatinine, previous coronary artery bypass grafting, tricuspid regurgitation and use of apixaban were independently associated with the occurrence of secondary endpoint (Table Supplement 4). Kaplan–Meier curves were also generated for secondary endpoint based on the anticoagulation prescribed at the time of discharge. This revealed a significant difference in the rates of secondary endpoint between groups (p < 0.001) (Fig. 3).

### Subgroup analysis

**Fig. 4:**
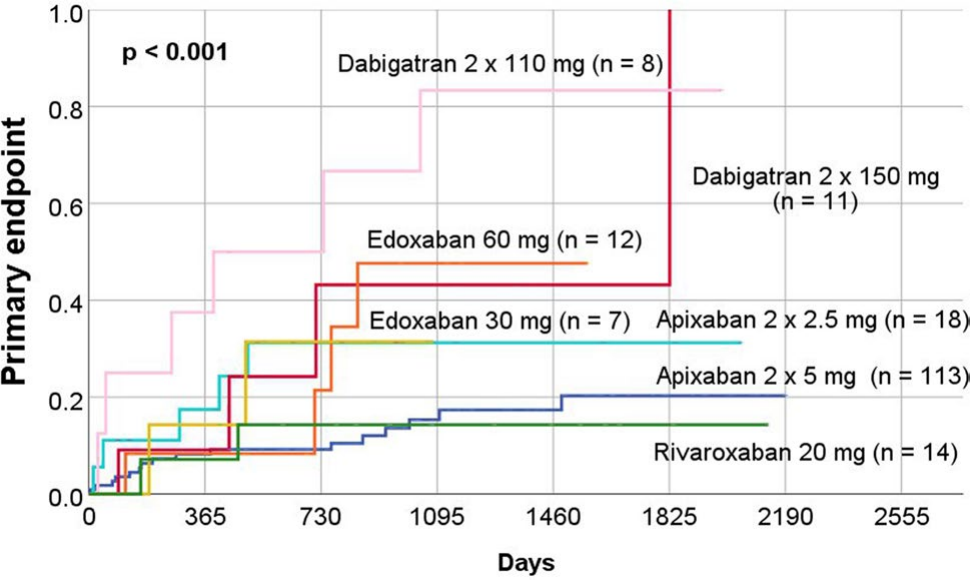
Long-term outcome (primary endpoint) in the study patients according to standard or reduced dose of oral anticoagulation at the time of discharge after initial transoesophageal echocardiography

In a subgroup analysis, we examined how dose reduction of DOAC affected rates of adverse events (primary endpoint). Figure 1 in the supplements illustrates the Kaplan–Meier curves of all patients who received a DOAC at discharge, either at the standard or reduced dose. Again, there were significant differences between the groups (*p* < 0.001) (Fig. 4).

**Fig. 5:**
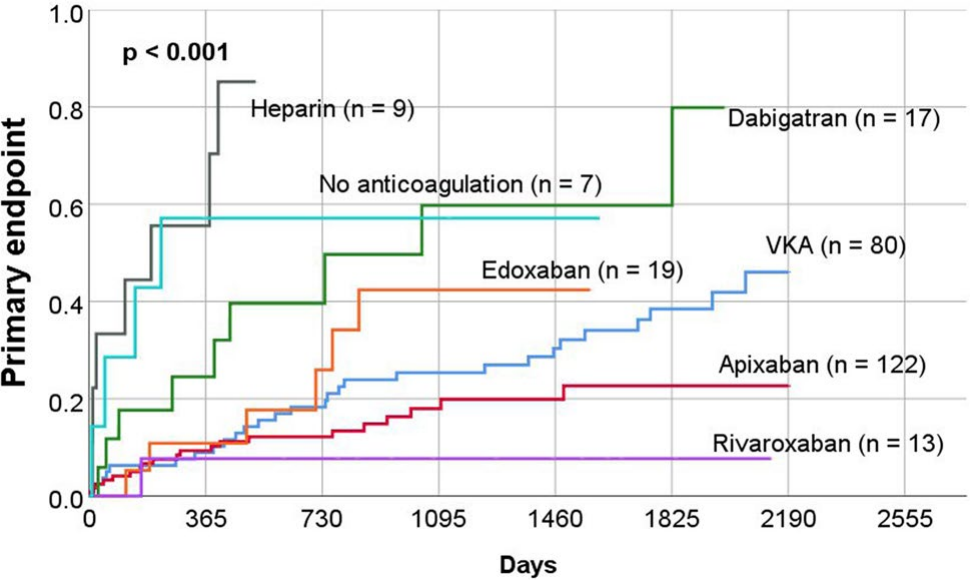
Long-term outcome (primary endpoint) subgroup of study patients with non-valvular atrial fibrillation according oral anticoagulation at the time of discharge after initial transoesophageal echocardiography

In a further subgroup analysis, only patients with AF without valve replacement and valve repair or mitral stenosis were included (“non-valvular AF”). The anticoagulation of the remaining 267 patients was also analyzed by Kaplan–Meier curves. Furthermore, there was a significant difference in patients` outcome (*p* < 0.001) (Fig. 5).

## Discussion

The present study investigated the outcome of patients with evidence of thrombus or SEC in the LAA on transoesophageal echocardiography in the era of DOAC. The study has several main findings. First, it should be noted that patients with thrombus did not differ significantly in clinical and echocardiographic characteristics compared with patients with SEC—except for left ventricular ejection fraction. The clinical outcome of patients with thrombus or SEC is similar, although patients with thrombus have a significantly higher event rate than patients with SEC (Fig. 1).

Independent factors for the occurrence of adverse events (primary endpoint: composite endpoint of all-cause mortality, non-fatal stroke or TIA, non-fatal systemic embolization, non-fatal major bleeding, and non-fatal myocardial infarction) in the long-term follow-up were age, haemoglobin, previous coronary artery grafting, dialysis and choice of anticoagulation.

### Incidence of thrombus and SEC in the LAA in the DOAC era

The exact prevalence of thrombi and SEC in the LAA is unclear. In a meta-analysis by Di Minno et al., the prevalence of thrombi in the left atrium including the LAA was described to be approximately 10% in patients with nonvalvular AF [24]. In a review by Patel et al., a frequency of SEC in patients with AF was reported to be 12–67% [25]. In contrast to the above publications, Vinereanu et al. reported a prevalence of 2% of thrombi and SEC in the left atrium on baseline echocardiography in a subgroup analysis of ARISTOTLE [17]. Recently, in a prospective multicenter registry of 6186 patients with AF undergoing radiofrequency catheter ablation on uninterrupted DOAC, no thrombus was detected on transoesophageal echocardiography, but SEC was detected in 27% [25]. By contrast, Wu et al. reported an incidence of 2.8% thrombi and 2.5% dense SEC in a similar study population with DOAC anticoagulated patients [26].

In our study, thrombus and SEC in the LAA were detected in 1.2% and 6.2%, respectively, of all transoesophageal echocardiographies that were performed in our hospital between 2015 and 2020. This means that in every 15th transoesophageal echocardiography, a finding associated with increased risk of stroke and poor prognosis was detected [2–7]. It is worth mentioning that in our study, anticoagulation with a DOAC was already used in one third of all patients when thrombi or SEC were detected. On the other hand, approximately 40% of all patients were not taking anticoagulation previously and the remaining patients were treated with VKA or heparin. It can be assumed that the proportion of DOAC will continue to increase in the future.

Impact of anticoagulation on event-free long-term survival.

Until recently, European guidelines recommended the use of vitamin K antagonists for at least 3 weeks to dissolve thrombi in the LAA and long-term therapy for persistent thrombin [27]. In the last years, first case reports and later smaller studies were published suggesting the usage of NOAC as a possible therapeutic option for thrombi or SEC [15].

Two prospective, multicentre trials demonstrated that rivaroxaban is effective in the treatment of LAA and left atrial thrombi. However, no long-term results were reported [18, 19]. In a subgroup analysis of the ARISTOTLE study, it was reported that patients on effective anticoagulation with apixaban or warfarin had no increased risk of thromboembolic events even in the presence of thrombi or SEC in the LAA [17].

Our study revealed differences in outcome according to the choice of anticoagulation (Figs. 2, 3 and 5). The use of heparin alone for anticoagulation was associated with worse outcome. This result can be explained by the fact that those medicated only with heparin were often patients who were seriously ill and treated in the intensive care unit for a longer period. Similarly, the non-anticoagulated group is heterogeneous and partly burdened with considerable comorbidities, which also explains the increased rate of events.

The multivariate analysis demonstrated that the prescription of apixaban at discharge was an independent factor for a lower rate of adverse events (Table 4). The group of patients prescribed rivaroxaban also had a low rate of adverse events. However, this subgroup included only a few patients, so there were no significant differences.

Similarly, the subgroup of patients who were prescribed edoxaban or dabigatran at discharge was small. Furthermore, it should be noted that our study was not able to investigate changes in oral anticoagulation or patient compliance. The results must therefore be interpreted with caution. Nevertheless, our study suggests that the use of dabigatran in patients with thrombi or SEC in the LAA may be unfavourable in the long-term. The observed differences in outcome may be explained by the different effects of the drugs [28].

### Limitations

The main limitation of the present study is the retrospective and monocentric character of the study. Therefore, it has not been feasible to evaluate the patients in terms of atrial hemodynamics or laboratory parameters, which may also have influenced the outcome. The choice of anticoagulation or further therapy (e.g. pulmonary vein isolation, cardioversion, interventional LAA closure, etc.) was at the discretion of the treating physicians and was not defined by a study protocol. This may have biased the study results. On the other hand, the approach described here may be more in line with clinical routine. In our study, we were also unable to investigate changes in prescribed anticoagulation or patient compliance. This may be particularly important in the long term. Furthermore, the subgroups of the different anticoagulants (in particular edoxaban, dagibatran and rivaroxaban) are partly small. The results of the present study with respect to the choice of anticoagulation should therefore be evaluated with caution. Nevertheless, the present study is the largest study that has ever investigated the long-term outcome of patients with thrombus and SEC in the LAA under different anticoagulants.

## Conclusion

Even in the era of DOAC, thrombus or SECs are detected in the LAA in more than 7% of all trans-esophageal echocardiographies. In the short term, the clinical outcome of patients with thrombus and SEC is poor, with thrombus being associated with a slightly more frequent proportion of adverse advents. Moreover, our study suggests that differences in the long-term outcome may depend on the choice of anticoagulation.

## Supplementary Information

Below is the link to the electronic supplementary material.

Supplementary file1 (DOCX 22 kb)

Supplementary file2 (DOCX 17 kb)

## Abbreviations

AF: Atrial fibrillation
LAA: Left atrial appendage
MACCE: Major adverse cardiac and cerebrovascular events
DOAC: Direct acting anticoagulants
SEC: Spontaneous echo contrast
TIA: Transient ischaemic attack
VKA: Vitamin K antagonist
