# Supplementary material for: Short- and long-term outcome of patients with spontaneous echo contrast or thrombus in the left atrial appendage in the era of the direct acting anticoagulants
Source: Clin Res Cardiol. 2021 Aug 26;110(11):1811–21. doi: 10.1007/s00392-021-01926-8 (PMC8563546; doi:10.1007/s00392-021-01926-8)
Supplement: Supplementary file 1 — Supplementary file1 (DOCX 22 kb) [file 392_2021_1926_MOESM1_ESM.docx]

**Tables**

**Table 1 Supplement:** Baseline Characteristics in patients with and without secondary endpoint (all-cause death) (n = 302)

|  | All-cause death  (n = 70) | No all-cause  death  (n = 233) | p value |
| --- | --- | --- | --- |
| Age (years) | 75.8 ± 7.8 | 70.9 ± 10 | < 0.001 |
| Women (♀), n (%) | 31 (44) | 102 (44) | 0.962 |
| Medical history |  |  |  |
| Hypertension, n (%) | 65 (93) | 211 (91) | 0.618 |
| Diabetes mellitus, n (%) | 30 (43) | 64 (27) | 0.016 |
| Atrial fibrillation, n (%) | 66 (94) | 225 (97) | 0.291 |
| Coronary artery disease, n (%) | 32 (46) | 66 (28) | 0.007 |
| Previous myocardial infarction, n (%) | 12 (17) | 28 (12) | 0.272 |
| Coronary artery bypass grafting, n (%) | 11 (16) | 7 (3) | < 0.001 |
| Previous stroke/TIA, n (%) | 17 (24) | 41 (18) | 0.224 |
| Peripheral artery disease, n (%) | 13 (19) | 28 (12) | 0.164 |
| Chronic obstructive lung disease, n (%) | 12 (17) | 21 (9) | 0.057 |
| Heart failure, n (%) | 34 (49) | 67 (29) | 0.002 |
| ICD/CRT, n (%) | 11 (16) | 9 (4) | <0.001 |
| Dialysis, n (%) | 6 (9) | 0 | < 0.001 |
| CHA2DS2-VASc Score (pts) | 4.8 ± 1.7 | 3.8 ± 1.7 | < 0.001 |
| Labor |  |  |  |
| Hemoglobin (g/dL) | 12 ± 2.2 | 13.8 ± 1.8 | < 0.001 |
| Creatinine (mg/dL) | 1.4 ± 1.32 | 1.04 ± 0.34 | < 0.001 |

TIA, transient ischaemic attack, ICD/CRT, implantable cardioverter defibrillator/ cardiac resynchronisation therapy

**Table 2 Supplement:** Transthoracic and transoesophageal echocardiographic parameters of study patients (n = 302)

|  | All-cause death  (n = 70) | No all-cause  death  (n = 233) | p value |
| --- | --- | --- | --- |
| Left ventricular ejection fraction (%) | 47.4 ± 11.6 | 48.6 ± 11.8 | 0.460 |
| Left atrial diameter (mm) | 46 ± 5.9 | 43.8 ± 5.4 | 0.006 |
| Ventricular septum thickness (mm) | 12.6 ± 2.1 | 12.2 ± 2 | 0.262 |
| Left atrial appendage thrombus, n (%) | 15 (21) | 36 (15) | 0.247 |
| Spontaneous echo contrast (mild-to moderate, severe, sludge) | 38/14/3 | 157/38/2 | 0.060 |
| Aortic stenosis (none/ mild/ moderate/ severe), n | 64/2/2/2 | 221/3/3/5 | 0.573 |
| Aortic regurgitation (none/ mild/ moderate/ severe), n | 28/39/1/3 | 140/85/7/0 | 0.003 |
| Mitral stenosis (none/ mild/ moderate/ severe), n | 68/1/1/0 | 225/4/1/2 | 0.696 |
| Mitral regurgitation (none/ mild/ moderate/ severe), n | 5/53/10/2 | 24/169/38/1 | 0.173 |
| Tricuspid regurgitation (none/ mild/ moderate/ severe), n | 16/38/13/3 | 82/130/18/3 | 0.012 |
| Aortic valve replacement/TAVI, n (%) | 6 (9) | 5 (2) | 0.012 |
| Mitral valve repair/ replacement n (%) | 2 (3) | 8 (3) | 0.809 |

TAVI, transaortic valve implantation

**Table 3 Supplement:** Medication at discharge after initial transoesophageal echocardiography of study patients (n = 302)

|  | All-cause  death  (n = 70) | No all-cause  death  (n = 233) | p value |
| --- | --- | --- | --- |
| Acetylsalicylic acid, n (%) | 16 (23) | 41 (18) | 0.331 |
| P2Y12-Inhibitor, n (%) | 2 (3) | 14 (6) | 0.298 |
| Dual therapy*, n (%) | 10 (14) | 29 (12) | 0.696 |
| Triple therapy*, n (%) | 0 | 10 (4) | 0.077 |
| Vitamin-K antagonist, n (%) | 29 (41) | 68 (29) | 0.057 |
| Apixaban, n (%) | 15 (21) | 116 (50) | < 0.001 |
| Dabigatran, n (%) | 7 (10) | 12 (5) | 0.145 |
| Edoxaban, n (%) | 5 (7) | 14 (6) | 0.738 |
| Rivaroxaban, n (%) | 1 (1) | 13 (6) | 0.145 |
| Heparine, n (%) | 7 (10) | 5 (2) | 0.003 |
| No anticoagulation, n (%) | 6 (9) | 4 (2) | 0.005 |

*Dual therapy, combination of Acetylsalicylic acid/P2Y12 and DOAC/ Vitamin-K antagonist, *Triple therapy, combination of Acetylsalicylic acid, P2Y12 and DOAC/ Vitamin-K antagonist. DOAC and a Vitamin K antagonist are not used concomitantly.
