# Supplementary material for: Short- and long-term outcome of patients with spontaneous echo contrast or thrombus in the left atrial appendage in the era of the direct acting anticoagulants
Source: Clin Res Cardiol. 2021 Aug 26;110(11):1811–21. doi: 10.1007/s00392-021-01926-8 (PMC8563546; doi:10.1007/s00392-021-01926-8)
Supplement: Supplementary file 2 — Supplementary file2 (DOCX 17 kb) [file 392_2021_1926_MOESM2_ESM.docx]

**Table 4 Supplement:** Univariate und multivariate analysis: predictors of long-term outcome

|  |  | Univariate analysis |  |  | Multivariate analysis |  |  |
| --- | --- | --- | --- | --- | --- | --- | --- |
|  | Hazard ratio | Confidence interval | p value | Hazard ratio | Confidence interval | p value |  |
| Age (years) | 1.071 | 1.038 – 1.105 | < 0.001 | 1.048 | 1.012 – 1.086 | 0.009 |  |
| Hemoglobin (g/dL) | 0.646 | 0.579 – 0.720 | < 0.001 | 0.695 | 0.618 – 0.783 | < 0.001 |  |
| Creatinine (mg/dL) | 2.158 | 1.697 – 2.745 | < 0.001 | 1.774 | 1.38 – 2.277 | < 0.001 |  |
| CHA2DS2-VASc Score (pts) | 1.368 | 1.295 – 1.562 | < 0.001 |  |  |  |  |
| Diabetes mellitus | 1.853 | 1.146 – 2.998 | 0.012 |  |  |  |  |
| Heart failure | 2.191 | 1.366 – 3.513 | 0.001 |  |  |  |  |
| Coronary artery disease | 1.744 | 1.085 – 2.803 | 0.022 |  |  |  |  |
| Coronary artery bypass grafting | 4.093 | 2.141 – 7.825 | < 0.001 | 2.728 | 1.389 – 5.361 | 0.004 |  |
| Dialysis | 13.766 | 5.836 – 32.469 | < 0.001 |  |  |  |  |
| Left atrial diameter | 1.080 | 1.031 - 1.131 | 0.001 |  |  |  |  |
| Aortic regurgitation | 1.629 | 1.139 – 2.331 | 0.008 |  |  |  |  |
| Tricuspid regurgitation | 1.743 | 1.243 – 2.443 | 0.001 | 1.564 | 1.078 – 2.268 | 0.018 |  |
| Aortic valve replacement | 3.173 | 1.371 – 7.342 | 0.007 |  |  |  |  |
| ICD/CRT | 3.919 | 2.041 – 7.522 | < 0.001 |  |  |  |  |
| Heparine | 6.918 | 3.087 – 15.506 | < 0.001 |  |  |  |  |
| Apixaban | 0.374 | 0.211 – 0.664 | 0.001 | 0.414 | 0.227 – 0.755 | 0.004 |  |
| No anticoagulation | 4.785 | 2.053 – 11.156 | < 0.001 |  |  |  |  |

TIA, transient ischaemic attack, ICD/CRT, implantable cardioverter defibrillator/ cardiac resynchronisation therapy
